# A short version of the post-COVID-19 condition stigma questionnaire

**DOI:** 10.1016/j.puhip.2025.100696

**Published:** 2025-12-11

**Authors:** Liam Rourke, Ronald Damant

**Affiliations:** a 15-757 Orwell Street, North Vancouver, British Columbia, V7J 3K6, Canada; b University of Alberta, Edmonton, Alberta, Canada

**Keywords:** Health-related stigma, Long COVID, Post-COVID-19 Condition

## Abstract

**Objectives:**

The purpose of this study was to develop a short version of the 40-item Post-COVID-19 Condition Stigma Questionnaire (PCCSQ) while preserving its factor structure, reliability, and validity. The PCCSQ is a sound tool for assessing the discrimination experienced by people with a diagnosis of long covid, but a shorter version would be less demanding of respondents experiencing fatigue and brain fog and easier for clinicians and researchers to administer.

**Study design:**

This was an observational study.

**Methods:**

From the original 40-items measuring the 6-factor construct *long covid stigma*, we assembled 12 items that represented the factors and discriminated among participants with high and low levels of stigma. We administered the shorter questionnaire to 99 long covid patients and assessed several of its measurement properties.

**Results:**

The 12-item instrument maintains the 6-factor structure of long covid stigma, has a mean discrimination index of 0.40 (sd = 0.08; range 0.22–0.48), an internal consistency of α = 0.89, a split-half reliability of 0.86, and it correlates predictably with theoretically-related variables.

**Conclusions:**

The PCCSQ-12 is a feasible, reliable and valid means of assessing patients’ experience of long covid stigma.

## 1 Introduction

Health-related stigma is the labeling, stereotyping, othering, and discrimination of people with diagnoses and symptoms of various health conditions. These conditions include mental health concerns [1], HIV [2], lung cancer [3], and others. Most recently, it has been experienced by people with post-COVID-19 condition or *long covid* [4,5]

An early survey of people with long covid [5] found that nearly 80 % of respondents experienced stigma *often* or *always*. Most often they experienced *anticipated stigma*, operationalized with questionnaire items such as, “I worry that people will judge me negatively when they learn I have long covid.” They also experienced *internalized stigma*, which was operationalized with items such as “I feel that I have been tainted by long covid and have less value than others”. Respondents also reported *enacted stigma*, defined as direct overt experiences of discrimination.

As this data was being gathered in the United Kingdom, we were engaged in a similar project in Canada [4]. We too found that participants agreed with questionnaire items that portrayed experiences of stereotyping, social and physical isolation, and self-stigma. We also demonstrated relationships between participants’ scores on our *post-COVID-19 Condition Stigma Questionnaire* (PCCSQ) and symptoms, functional status, and health-related quality of life.

The PCCSQ consists of 40-items representing 6 dimensions of long covid stigma (negative self-image, social isolation, disclosure concerns, fear of PCC, COVID-19 stereotyping, and concerns with public attitudes), This is considerably more items than other instruments use to measure health related stigmas. The HIV Stigma Questionnaire [6], for instance, which is the template for many subsequent stigma questionnaires, measures its construct reliably and validly with 12 items. Superfluous items are a particular concern with our population whose most common symptoms include difficulties concentrating, memory loss, and tiredness [7]. Excessively lengthy instruments are also a burden for health researchers whose studies often include multiple instruments [8], with each increasing the strain on recruitment and analysis. Irrespective of these concerns, researchers consistently find that longer questionnaires are associated with diminished response quality [9].

The purpose of this study was to shorten the PCCSQ while maintaining its factor structure, reliability, and validity.

## 2 Methods

This study drew on a set of data and analyses conducted during our development of the 40-item PCCSQ. The data was gathered from a convenience sample of 145 patients recruited from a long covid clinic in Alberta Canada. Their mean age was 48.2 (sd = 12.2), and 66.2 % identified as female. One-hundred and eight (74.5 %) were white; 28 (19.3 %) were non-white, non-indigenous; 8 (5.5 %) identified as Indigenous (First Nations/Inuit/Metis). Body Mass Index (BMI) ranged from 18.3 to 60.5 kg/m^2^, with a mean (SD) of 31.7 (8.3).A full description of that project is available [4]. To build a 12-item version of the questionnaire, we.

● Calculated a discrimination index for all 40 items and selected two from each factor that most clearly discriminated between respondents with high (upper third) and low (lower third) stigma scores.
● Assembled these items into a 12-item version of the questionnaire (PCCSQ-12):
  1. Most people are uncomfortable around someone with my health problems.
  2. Stereotypes about people with my health problems are true.
  3. I worry that people will discriminate against me because of my health problems.
  4. I have been hurt by how people react to my health problems.
  5. In general, telling someone about my health problems is a mistake.
  6. People don't want their children to be around me because of my health problems.
  7. I have stopped socializing with some people because of my health problems.
  8. People seem afraid of me once they learn about my health problems.
  9. People are afraid of someone who has post-acute or long COVID-19 (recovered from the acute COVID-19 infection, no longer on isolation, persistent non-infectious health problems).
  10. People treat me like I have an infectious (contagious) disease.
  11. I feel isolated from the rest of the world because of my health problems.
  12. Telling someone about my health problems is risky.

(Responses are registered with a 5-point Likert-style scale anchored at either end by *Disagree strongly* and *Agree strongly.*)

● Administered the PCCSQ-12 to 99 participants from the original study (1.6 years after the initial administration), and from their responses, calculated a total stigma score (TSS-12), Cronbach's alpha, split-half reliability, and correlations between TSS-12 and seven health variables theoretically-related to the long covid stigma. These variables (and the instruments used to measure them) were: post-COVID-19 functional status (post-COVID-19 functional status scale), depression, anxiety, and number of symptoms (Edmonton Symptom Assessment Scale-Revised), symptom burden (participants' ratings of the ESAS items on a visual analogue scale), and health-related quality of life (Patient Health Questionnaire-9 and EQ 5D5L).

## 3 Results

The mean of the discrimination indices for all 12 items was 0.40 (sd = 0.08; range 0.22–0.48). Discrimination indices of 0.40 and above are generally considered to discriminate effectively between groups.

The internal consistency of the scale was evaluated using Cronbach's alpha, which yielded a coefficient of α = 0.89. This indicates a high level of internal consistency among the items. The split-half reliability for the questionnaire was 0.86 indicating a strong positive relationship between the two halves.

Correlations between the PCCSQ-12 and theoretically-relevant variables ranged from r = 0.40 to r = 0.73, all of which were significant p < 0.001: Post-COVID-19 Functional Status r = 0.59, p < 0.001; number of symptoms r = 0.60, p < 0.001, symptom burden r = 0.63, p < 0.001, ESAS-r depression r = 0.45, p < 0.001, ESAS-r anxiety r = 0.40, p < 0.001; PHQ-9 r = 0.42, p < 0.001; EQ 5D5L r = 0.73, p < 0.001.

## 4 Discussion

The purpose of this study was to build a version of the PCCSQ that is less demanding on respondents and easier for clinicians and researchers to administer. The PCCSQ-12 is 70 % shorter than the original instrument; yet it maintains the latent six-factor structure of long covid stigma and yields estimates of respondents’ experiences that, provisionally, are reliable and valid.

Our findings are similar to those of others who have shortened questionnaires designed to measure various forms of health related stigma. Shortened versions of the HIV-Stigma Questionnaire and the Lung Cancer Stigma Scale have psychometric properties comparable to their originals’, and they have increased the volume of research on both forms of health-related stigma.

Our conclusions about the reliability and validity of the PCCSQ-12 derive from its use with one convenience sample of volunteers, so they are limited. Successive administrations would support or contradict our assessment of the soundness of interpretations that arise from participants’ responses.

Regarding suggestions for practice and research, we point to the strong relationship between the PCCSQ-12, health outcomes, and general well-being. Administering the questionnaire more widely could produce insights into the confounding biomedical phenomena of long covid and into the broader social phenomenon of stigma.

## Ethical statement

The protocol for our original research and follow up studies were approved by the University of Alberta Research Ethics Board (Pro 00107350).

## Funding

The authors wish to acknowledge the Long COVID Web for its role in supporting the completion of this Research Project. The Long COVID Web is funded by the Canadian Institutes of Health Research (CIHR) grant #185352.

## Declaration of competing interest

We declare that we have no conflicts of interest related to this research, including any financial, professional, or personal affiliations that could influence the collection, analysis, or reporting of data. We commit to maintaining transparency and adhering to institutional policies for the management of conflicts of interest.
